# Short photoperiod modulates behavior, cognition and hippocampal neurogenesis in male Japanese quail

**DOI:** 10.1038/s41598-023-28248-1

**Published:** 2023-01-18

**Authors:** Marion Georgelin, Vitor Hugo Bessa Ferreira, Fabien Cornilleau, Maryse Meurisse, Kévin Poissenot, Massimiliano Beltramo, Matthieu Keller, Léa Lansade, Hugues Dardente, Ludovic Calandreau

**Affiliations:** CNRS, IFCE, INRAE, UMR 85 Physiologie de la Reproduction et des Comportements, Université de Tours, PRC, 37380 Nouzilly, France

**Keywords:** Animal behaviour, Animal physiology, Cognitive neuroscience, Emotion, Learning and memory, Neurogenesis

## Abstract

The mechanisms underlying the photoperiodic control of reproduction in mammals and birds have been recently clarified. In contrast, the potential impact of photoperiod on more complex, integrative processes, such as cognitive behaviors, remains poorly characterized. Here, we investigated the impact of contrasted long and short photoperiods (LP, 16 h light/day and SP, 8 h light/day, respectively) on learning, spatial orientation abilities, and emotional reactivity in male Japanese quail. In addition, we quantified cell proliferation and young cell maturation/migration within the hippocampus, a brain region involved in spatial orientation. Our study reveals that, in male quail, SP increases emotional responses and spatial orientation abilities, compared to LP. Behaviorally, SP birds were found to be more fearful than LP birds, exhibiting more freezing in the open field and taking longer to exit the dark compartment in the emergence test. Furthermore, SP birds were significantly less aggressive than LP birds in a mirror test. Cognitively, SP birds were slower to habituate and learn a spatial orientation task compared to LP birds. However, during a recall test, SP birds performed better than LP birds. From a neuroanatomical standpoint, SP birds had a significantly lower density of young neurons, and also tended to have a lower density of mature neurons within the hippocampus, compared to LP birds. In conclusion, our data reveal that, beyond breeding control, photoperiod also exerts a profound influence on behavior, cognition, and brain plasticity, which comprise the seasonal program of this species.

## Introduction

To survive and reproduce efficiently, organisms constantly need to be well synchronized with environmental cues, and adjust different aspects of their biology, such as behavior, physiology, and neurobiology, to potential changes that may occur in their surroundings^1^. An important environmental cue that may impact physiology of both human and non-human animals is photoperiod (day length)^1–3^. Because of its stability and predictability throughout the calendar year, photoperiod is the key coordinator of seasonal adaptations, at least at temperate and polar latitudes^4,5^. Indeed, changes in photoperiod trigger many physiological and behavioral adaptations (e.g., reproduction, migration, hibernation) that ultimately allow an optimal fit of the individual to its current living conditions^1,6^.

While the mechanisms of reproductive regulation by photoperiod in mammals and birds have been widely described in the scientific literature^4,7^, the impacts of photoperiod on more complex, integrative functions, such as cognitive behaviors, are still poorly characterized^8^. For instance, photoperiod can profoundly alter how animals perceive, interpret, and memorize information from their environment. At the emotional/behavioral level, short photoperiod intensifies conditioned fear responses in rodents: white-footed mice (*Peromyscus leucopus*) reared in short photoperiod (SP; 8 h light/day) display a stronger fear conditioning response between a sound and a negative stimulus (i.e., an electric shock delivered to the animal's paws) compared to individuals reared in long photoperiod (LP; 16 h light/day)^9^. Similarly, Siberian hamsters (*Phodopus sungorus*) reared in SP expressed more depressive-like behaviors during a forced swimming test compared to individuals reared in LP^10^.

Another impact of photoperiod is observed at the cognitive level: SP-reared white-footed mice display lower spatial learning and memory performance (i.e., greater latency to find the location of an immersed platform in a water maze) than individuals reared in LP^11^. This difference in spatial memory is mirrored by neuroanatomical changes. Compared to LP-exposed animals, the hippocampus of SP-exposed animals had fewer new-born neurons, a lower density of dendritic spines, and altered synaptic efficiency of hippocampal cells^11,12^. Intriguingly, opposite results have been reported in some bird species. For example, marsh tits (*Poecile palustris*) performed better in a spatial memory test under SP than under LP^13^ and the number of newly formed neurons in the hippocampus, was greater during SP than LP in great tits (*Parus major*)^14^. However, a potential confounding factor in these studies is the use of individuals taken from their natural environment and placed in captivity. Captivity is a well-known stress factor that can induce strong behavioral and cognitive disturbances. It is therefore possible that the observed differences are due, at least in part, to stress-related effects of captivity rather than to photoperiodic changes. These concerns remain to be addressed^15^.

Overall, exposure to SP appears to be associated with variation in learning and spatial orientation performance, and exacerbation of emotional responses. However, current literature on this topic is mostly focused on mammals, with only scarce, and mostly contrasting, data on avian species. To address this issue, we here focused on the impact of photoperiod on emotion, cognition, and neurobiology in male Japanese quail (*Coturnix coturnix*), a well characterized animal model, which has been extensively used over the last 50 years to identify the endocrine and molecular underpinnings of the photoperiod control of seasonal breeding^16–21^. More precisely, we investigated the effects of exposure to LP (16 h light/day) and SP (8 h light/day) on learning, spatial orientation abilities, and emotional reactivity in different behavioral tests (open field, mirror, and emergence tests). Since hippocampal neurogenesis is involved in learning and spatial orientation^22–25^ and appears to be sensitive to photoperiod, we also tested the impact of photoperiod on hippocampal neurogenesis in some of these birds. We predicted that, similar to what was found for the marsh and great tits^13,14^, exposing quail to SP would increase their learning and spatial orientation abilities. Our previous studies in quail showed that fearful quail have better spatial abilities than less fearful individuals^25,26^. Based on this, it could be anticipated that SP might increase emotional reactivity of quail as well. Contrary to the marsh and great tit studies, and based on our own previous results in “highly emotional” quail, we further predicted that exposing quail to SP would decrease cell proliferation and maturation/migration in the hippocampus^25^.

## Materials and methods

### Ethics statement

Experiments were conducted using Japanese quail (*Coturnix japonica*) bred and maintained in our research facilities since 1980. Parental birds originated from two commercial lines (a meat-type, broiler line and a cross between a broiler and a layer line) that were reciprocally crossed and had their progeny divergently selected for either duration of tonic immobility (TI, aimed to study bird fearfulness) or social reinstatement behavior (aimed to study bird sociability). For this experiment, we used the 61^st^ generation of the unselected, control TI line, characterized by birds that presented an intermediate duration of tonic immobility, compared to their selected counterparts. For further details concerning the historical background of these quail lines, see^27^.

The whole experiment took place at the Pôle d’Expérimentation Avicole de Tours (UE PEAT, INRAE, 2018. Experimental Poultry Facility, https://doi.org/10.15454/1.5572326250887292E12). Animal care and experimental treatments followed the French Ministry of Agriculture guidelines and European regulations on animal experimentation (86/609/EEC). They also followed local animal regulations (C37-175-1) of the French Ministry of Agriculture under the EEC directive and under the INRAE ethics committee approval (agreements N° 1789 and 1848, Val de Loire). This study was carried out in compliance with the ARRIVE guidelines.

### Animals

After hatching, quail were placed in a warm communal pen (39 °C), where food and water were provided ad libitum. For the first three days of age (from d1 to d3), quail were reared under continuous light and then the light program was gradually switched to LP (16 L:8 D). The temperature was gradually decreased to reach 21 °C at d21. At this age, quail were also sexed and only males were used for experiments. Males were favored, over females, in these experiments because their degree of sexual development is strongly related to photoperiod and has already been well documented in the scientific literature: on the one hand, an increase in cloacal cleft diameter, proctodeal gland, and testicular volume is observed when animals are placed under long photoperiod conditions^18,19,28,29^. The cloacal protrusion, in males, serves as a convenient external index of androgen levels, allowing for rapid, reliable, and pain-free repeated measurements^19^. On the other hand, switching from LP to SP is associated with a regression of the cloacal cleft and testicles^28,29^.

At d21, male quail were randomly divided in two groups (n = 16 per group) and placed in different rooms. Animals were housed in individual cages in experimental batteries. Each cage (L 35 cm × W 24.5 cm × H 20.5 cm) had metal bar walls allowing for visual conspecific interactions to reduce social isolation. The cage floor was equipped with a plastic coating to reduce discomfort associated with the wire mesh surface. Artificial turf was also available on the cage floor, allowing animals to dust bathe. Unless otherwise stated, food and water were available ad libitum for each individual quail. The LP was maintained until d42 so that all the individuals were sexually fully mature.

From d42 and until the end of the experiment, one group was subjected to an SP transfer (8 L:16 D), while the other group remained in LP. To verify the impact of photoperiod in quail anatomical parameters, monitoring of bird growth was done once a week, three weeks before the photoperiodic transfer, and then, for the whole duration of the study. From d49 onwards, sexual development was also evaluated during every weighing, by measuring the diameter of the cloacal cleft (in centimeters), as a proxy for testicular development^30^.

**Figure 1:**
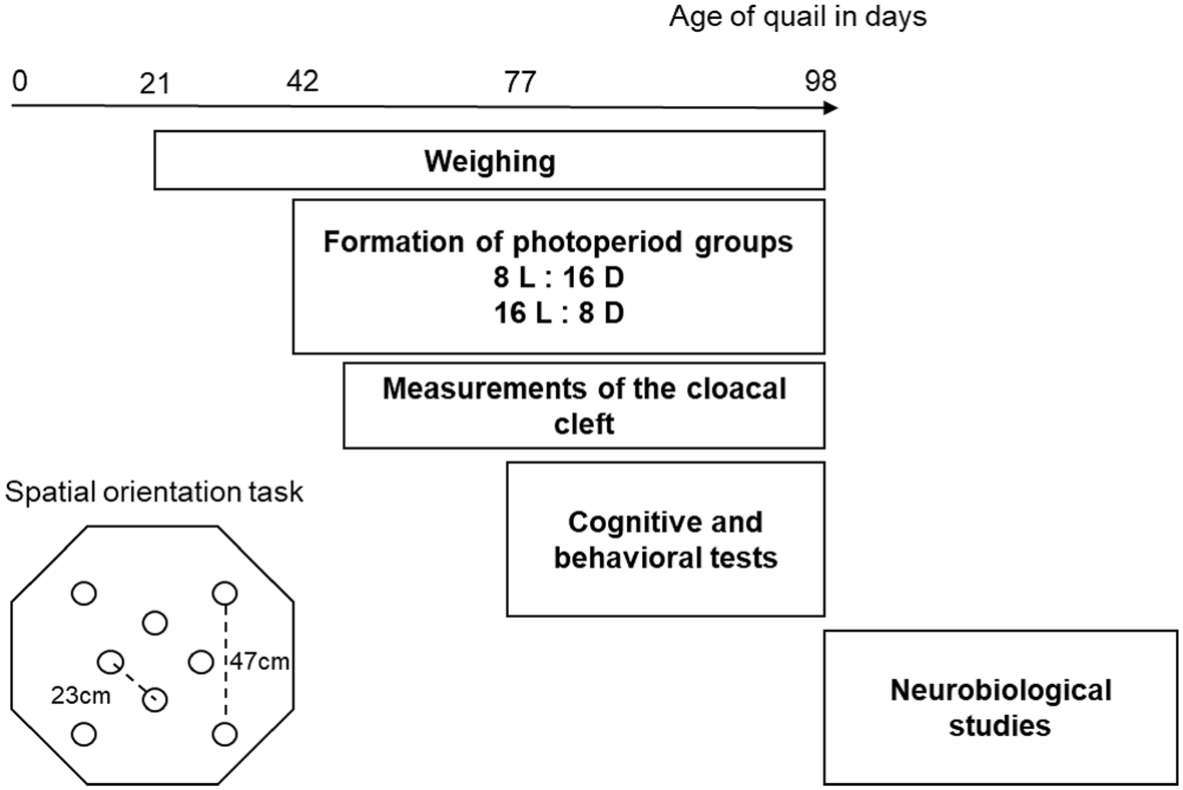
Schedule of the experiment and schematic representation of the apparatus used for the spatial orientation task. At 42-day old, quail were submitted to photoperiodic changes that lasted for the whole experiment. At 77-day old, short and long photoperiod birds (SP and LP, respectively) were trained in a spatial orientation task. At the end of the cognitive testing, animals were submitted to three different behavioral tests, namely, an open field test, a mirror test, and an emergence test. At the end of the experiment, some of the quail were euthanized and their brains were collected for immunohistochemical analyses.

At d77, the cognitive and behavioral tests started. Finally, at d98, the quail were euthanized, their brains removed and frozen for immunohistochemical analyses. The general schedule for the whole experiment is described in Fig. 1.

### Cognitive and behavioral tests

All following procedures, including the use of dedicated test arenas were previously validated by our group in both chicken and quail^24,26,31–35^.

After 5 weeks of exposure to either SP or LP, at the age of 11 weeks (d77), 16 birds of each group were submitted to a spatial orientation task and three behavioral tests (open field, mirror, and emergence tests). During the spatial orientation task, birds had to learn the spatial position of a target cup among eight identical cups placed in an octagonal arena. At the end of the cognitive testing, open field, mirror, and emergence tests were used to assess the emotional and behavioral state of birds. All behavioral data were recorded by a camera placed above the arenas and computerized by a tracking video system (Ethovision XT; Noldus IT, The Netherlands).

#### Spatial orientation task

##### Habituation

Each bird was individually placed in an octagonal arena (120 cm largest diameter; 50 cm side length) surrounded by a blue curtain (1.90 m high) to prevent the animal from escaping. A beige linoleum covered the floor of the arena, and the arena was lighted by a bulb at the ceiling (70 lx). Four black visual cues were placed on the walls of the arena and four others on the curtain. Eight identical opaque ceramic cups were placed in the arena^24–26,35^.

Before each habituation session (~ 4 h), food was removed from the home cage of the birds. Once daily, and for three consecutive days (d77–d79), each quail was placed into the center of the arena. All cups within the arena contained a mealworm. The mealworms were visible in the cups only from a short distance; therefore, the birds needed to approach the cups one by one to see their contents. The bird could freely explore the arena either until all cups were visited and all mealworms eaten, or for a maximum duration time of 600 s. On each occasion, the number of cups visited was scored^24–26,35^. Only individuals that visited at least five cups on the last day of habituation were retained for the training phase.

##### Training

Over eight consecutive days (from d80 to d87), birds were submitted to three training trials per day, spaced by one hour. As during habituation, the animals were fasted 4 h before their daily training started.

On each trial, the individual had to reach the location of a unique cup that was rewarded (henceforth ‘target cup’, rewarded with 4–5 mealworms). The other seven cups were identical to the target cup but did not contain a reward. The location of the target cup was constant across all days and trials, and it was the same for all individuals. The tested bird was introduced into the arena at three different random starting points. The trial ended when the tested bird reached the target cup and ate the mealworms or after a maximum test duration of 300 s, whichever came first. In order to keep the individual motivated, when it was not able to reach the target cup, the experimenter gently guided the animal to the target cup and allowed it to eat the mealworms, before removing it from the arena. Between each trial, the bird was returned to its home cage^24–26,35^.

On each trial, the latency to reach the target cup was recorded. If the target cup was not visited, the maximum latency (300 s) was assigned. The number of cups visited before reaching the target cup was also recorded. If the target cup was not visited, a score of eight, corresponding to the total number of cups in the arena, was assigned.

##### Probe test

The probe test was used to assess whether animals relied on their memory to locate the rewarded cup or used olfactory cues emanating from mealworms instead. On d88, animals were feed-restricted for at least one hour before their probe test. During the probe test all cups were empty. Each bird was introduced in the arena (random starting point) and allowed to freely explore the arena and the eight cups for 2 min. Similar to the training phase, both the number of cups visited before reaching the target cup (the cup rewarded during training) and the latency to reach it were recorded^24,26^.

#### Behavioral tests

##### Open field

Reactivity to a new environment^25,33,34^ was assessed on d92 in a square arena (0.8 × 0.8 × 0.7 m) of white wood with a floor made of beige linoleum. The arena was placed into an unknown experimental room, surrounded by an unknown white curtain and a very bright (50 lx) light illuminated the arena. Each quail was individually placed into the center of the arena and allowed to explore it for 5 min. The recorded behavior was the distance travelled within the arena.

##### Mirror test

One day after the open field test (on d93), and using the open field arena again, we tested quail aggressiveness^32^. A mirror (0.41 × 0.24 m) was sticked along one the sides of the arenas. The quail was gently placed in the center of the half side of the arena opposite to the mirror and facing the mirror. The quail was allowed to interact with its reflection for 5 min. The observed behaviors were time spent near the mirror (distance ≤ 20 cm), and the number of pecks against it.

##### Emergence

Finally, on d94, to test quail boldness and exploratory tendencies, we used a well-established emergence test^34,36^. The test arena was a square open space with wooden walls and a paper floor (0.62 × 0.6 × 0.33 m). At first, each bird was placed for 1 min in a closed and dark box (0.18 × 0.18 × 0.18 m) at one end of the corridor. After this time, a guillotine door was opened so that the animal had free access to the corridor for 5 min. The recorded behavior was latency to exit the box (full body out of the box).

### Immunohistochemistry

**Figure 2:**
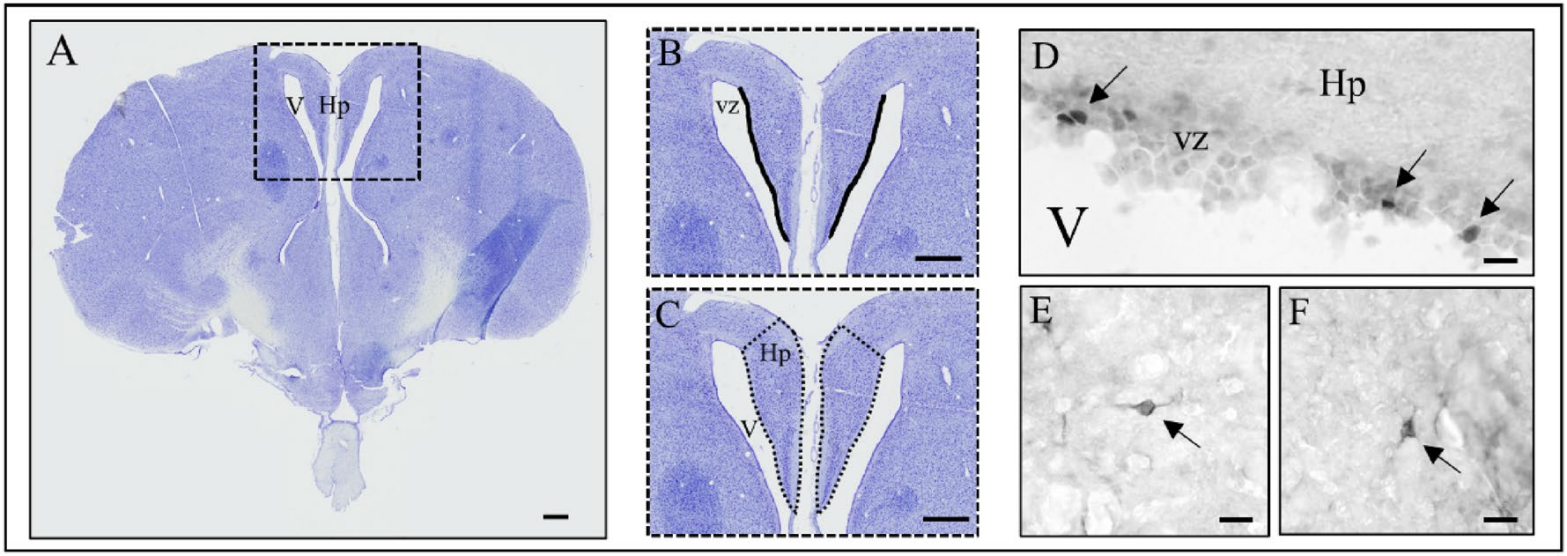
Neurobiological analysis. Scanned quail brain histological section stained with cresyl violet (**A**), with the detail of the counted areas: ventricular zone (vz, black line) of the hippocampus (**B**) and hippocampus (Hp, delimited by dotted lines; V for ventricle) (**C**). High magnification micrographs of PCNA (Proliferative Cell Nuclear Antigen) immunoreactive cells (**D**) and DCX (doublecortin) immunoreactive cells, bipolar immature (E) and multipolar mature (**F**). Arrows indicate specifically labelled cells. Scale bars: 500 µm (**A**–**C**), 10 µm (**D**–**F**).

For immunohistochemical analyses, birds were anesthetized at d98 with an overdose of pentobarbital 6% (36 mg/ 100 g). They were transcardially perfused with 80 mL 1% sodium nitrite in phosphate-buffered saline, followed by 300 mL of ice-cold 4% paraformaldehyde solution in 0.1 M phosphate buffer containing 15% (v/v) saturated picric acid. Brains were then removed from the skull, post-fixed overnight in the same fixative, cryoprotected in a 20% sucrose solution, frozen in isopentane at − 40 °C and stored at − 80 °C. Coronal sections were cut on a cryostat at a thickness of 25 µm. Six series of alternate sections (with an equal distance of 150 µm to one another) were collected onto SuperFrost® glass slides. One series was stained with Cresyl Violet to provide accurate anatomical localization (Fig. 2A). The other series were stored at − 80 °C for immunohistochemistry analysis. Immunohistochemistry was done on 4 –7 brains per group.

#### Cell proliferation

To assess cell proliferation in the ventricular zone of the hippocampus (Fig. 2B,C), we used immunolabeling for the endogenous marker Proliferative Cell Nuclear Antigen (PCNA) that was already validated in previous studies^25,37^. For this marker, an antigen retrieval was performed. After thawing, the slides were immersed for 10 min in a bath containing citrate buffer 0.01 M pH 6, at room temperature (RT). They were then warmed up to 85 °C in a microwave oven. After cooling to RT, the slices were rinsed three times for 5 min in TBS-0.3% Triton (TBST) and pre-incubated in blocking buffer (TBST-1‰ sodium azide (A) – Bovine Serum Albumin (BSA) 0.1%), for 10 min at RT. Then the sections were incubated for 24 h at 4 °C in the 1/500 anti-PCNA antibody (mouse anti-PCNA clone PC10; Leica Biosystems Cat# NCL-PCNA, RRID: AB_563951) in TBSTA-BSA 1%. After several rinses, sections were incubated in the 1/800 peroxidase-coupled secondary antibody (donkey anti-mouse; Jackson ImmunoResearch Labs Cat# 715-035-150, RRID: AB_2340770), in TBS-BSA, for 1.5 h at RT. After two rinses in TBST and two rinses in Tris-HCl, sections were treated for peroxidase activity detection with a mixture of 0.004% diaminobenzidine (Sigma-Aldrich, L’Isle d’Abeau Chesnes, France), 0.3% nickel ammonium sulphate, and 0.003% H_2_O_2_ solution in Tris-HCl (0.05 M, pH 7.6). The revelation was stopped by at least three successive rinses in Tris-HCl at 4 °C. All sections were finally mounted on gelatin-coated glass slides, air-dried, dehydrated in successive alcohol and toluene solutions, then coverslipped in Depex® (BDH Laboratory Supplies, UK).

#### Young cell maturation/migration

To assess young cell maturation/migration in the hippocampus, we used DCX (doublecortin) immunolabeling. To increase tissue permeabilization, sections were first treated in acetone for 15 min at − 20 °C. After being air-dried for 5 min, sections were treated in TBSTA-rabbit serum 1% for one hour at RT. Then, sections were incubated for two days at 4 °C in 1:300 anti-DCX primary antibody (goat anti-DCX, Santa Cruz Biotechnology Cat# sc-8066, RRID: AB_2088494) in TBSTA-rabbit serum 1%. Sections were then rinsed three times, ten minutes each, in TBST before being incubated for 3 h at room temperature in 1:300 secondary antibody (rabbit anti-goat-peroxidase; Jackson ImmunoResearch Labs Cat# 305-035-003, RRID: AB_2339400) in TBST-rabbit serum 1%. The protocol of the peroxidase detection was the same as for PCNA.

### Staining quantification

The density of positive cells (PCNA + and DCX + cells) was assessed using a motorized microscope (Axioskope 2; Zeiss, Germany) connected to the image analysis software Mercator® (Exploranova, La Rochelle, France) through a camera. Briefly, the sections chosen along the rostrocaudal axis were representative of the hippocampus and its ventricular zone. After drawing the hippocampus and its ventricular zone, Mercator® provided the measurement of the surfaces in mm^2^. After counting in the different areas, we then divided the number of PCNA and DCX positive cells by the surface (area) of the layer, resulting in the variables "Mean number of PCNA + cells / mm^2^" and "Mean number of DCX + cells / mm^2^".

The hippocampus (left and right sides) was drawn at a magnification of X10 and counting of PCNA and DCX immunoreactive cells was realized at a magnification of X20 (Fig. 2D–F). Quantification was done on both hemispheres. A scalpel mark was made in the right hemisphere of each brain. The principle of counting immuno-positive cells is based on the measurement of gray levels of cell marking. For both markers, we chose a strict thresholding criterion (around 70 on a gray scale ranging from 0 to 225), in order to unambiguously differentiate labeled cells from background noise. Counting is then done manually by pointing each and every immuno-positive cell. This method is more reliable than automatic counting because it takes into account potential slice artifacts (e.g., folding). The delimitation of the counting zones is carried out using a section stained with cresyl violet, adjacent to the immuno-stained section, which makes it possible to locate the different structures of interest. Brain structures were identified based on the atlases of the quail^38^ and chicken brain^39^, and we used the nomenclature adopted by the avian brain forum^40^. At the end of the counting session, we obtained the number of positive cells per surface of the area considered and deduced the density of immunolabeled cells. The counter was blind to the treatment.

For the counting of PCNA, 7–8 sections/animal were analyzed, with 300 µm between sections. PCNA + cells were counted into the ventricular zone adjacent to the left and right hippocampus. The thickness of the area bordering the ventricle (VZ) was about 25–30 µm and its length varied according to the antero-posterior level of the sections examined (mean length for the hippocampus 1.5 mm). For the manual counting of PCNA immune-positive cells we adjusted brightness and contrast to have the best discrimination between cells and background. The same settings were kept for all sections. Only black, round or ovoid stains were counted as positive nuclei.

For the counting of DCX, 5–6 sections/animal were analyzed, with 300 µm between sections. DCX is expressed by migrating neuroblasts and differentiating neurons. The two types of DCX + cells were counted in the hippocampus, in both hemispheres. Immatures neuroblasts were bipolar or fusiform (called « young-immunoreactive DCX cells »), and differentiating neurons were round multipolar (called « mature-immunoreactive DCX cells »). For the manual counting of DCX immune-positive cells we adjusted brightness and contrast to achieve optimal discrimination between cells and background. The same settings were kept for all sections. Cells were counted as positive when they presented black fibers and/or a clear unstained nucleus surrounded by a black cytoplasm.

### Statistical analysis

Our repeated response variables were as follows: weight of birds and diameter of the cloacal cleft; number of mealworms eaten during habituation for the spatial orientation task; latency to find the target cup, and number of cups visited before reaching the target cup (only the last two days of training to ensure birds from both treatments were at the same level before the probe trial) during training. A general linear model with repeated measures (ANOVA) was performed on these variables with photoperiod as between-subject factor, and weeks of age/task days as within-subject factors. For all ANOVA analyses, Greenhouse-Geisser corrections were applied when the assumptions of sphericity were violated. When main effects or interactions were significant, analyses were followed by multiple comparisons corrected by Bonferroni.

The variables ‘latency to find the target cup’, and ‘number of cups visited before reaching the target cup’ during probe trials, did not meet the assumptions for parametric statistics, even after transformations were done. Therefore, a non-parametric Mann–Whitney U test with Monte Carlo simulation was used to compare differences in each of these variables between our two photoperiodic groups.

Six SP birds failed to visit and eat the mealworms of at least five cups during the habituation phase; these birds were thus excluded from all cognitive analyses. Our final sample for the spatial orientation task was comprised of 10 birds for SP and 16 birds for LP.

Similar to probe trial variables, most of the variables from the remaining behavioral tests (open-field, mirror and emergence tests) did not meet the assumptions for parametric statistics, even after transformations were done. To keep consistency on the analyses of these variables, Mann–Whitney U test with Monte Carlo simulation was used to compare differences in each of these variables between our two photoperiodic groups (16 birds for both SP and LP).

Since birds have lateralized brains^41,42^, the mean densities of PCNA-immunoreactive cells and DCX-immunoreactive cells (young and mature ones) in the hippocampus were analyzed in the right hemisphere, left hemisphere, and both hemispheres combined (Total). Due to technical problems that reduced our sample size, a Mann–Whitney U test with Monte Carlo simulation was used to compare differences in each of these neurological variables between our two photoperiodic groups (PCNA cells: 4 birds in SP and 6 birds in LP DCX cells: 5 birds in SP and 7 birds in LP).

All statistical analyses were performed using IBM SPSS 21 and R v. 3.6.1. Statistical significance threshold was set at *p* ≤ 0.05. All data generated during this experiment can be accessed in the Supplementary material.

## Results

### Monitoring of individual growth and sexual development

**Figure 3:**
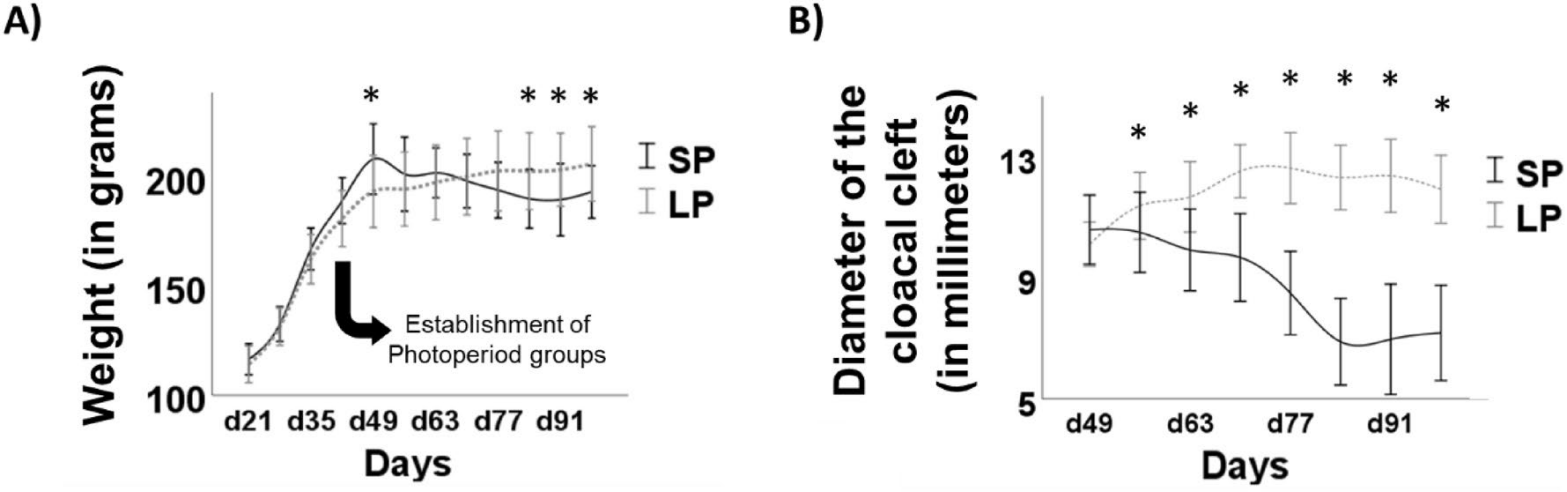
Effect of photoperiod in quail anatomical parameters. (**A**) Weight (in grams) of quail exposed to short (SP) and long (LP) photoperiod conditions. (**B**) Diameter of the cloacal cleft (in millimeters) for SP and LP birds. *p < 0.05, significant difference between short-photoperiod exposed birds and long-photoperiod exposed birds. Data are presented as mean ± S.D.

Growth was significantly different between the two photoperiod groups over weeks of age (effect of week: F_3.343, 100.305_ = 407.925, *p* < 0.001, ηp^2^ = 0.931; effect of photoperiod group: F_1, 30_ = 0.024, *p* = 0.877, ηp^2^ = 0.001; effect of week x photoperiod group: F_3.343, 100.305_ = 10.474, *p* < 0.001, ηp^2^ = 0.259, Fig. 3A). Post-hoc analyses revealed that, one week after the photoperiod switch, SP birds were heavier than LP birds (*p* = 0.016), but over the weeks, this tendency was reversed: LP birds became heavier than SP birds over the three last weighing (*p* = 0.03; *p* = 0.027; *p* = 0.02, respectively).

Similar to individual growth, sexual development also differed over time and photoperiod regimens (effect of week: F_4.779, 143.356_ = 13.589, *p* < 0.001, ηp^2^ = 0.312; effect of photoperiod group: F_1, 30_ = 96.75, *p* < 0.001, ηp^2^ = 0.763; effect of week × photoperiod group: F_4.779, 143.356_ = 41.295, *p* < 0.001, ηp^2^ = 0.579, Fig. 3B). During the first week after the photoperiod switch, there was no difference in cloacal cleft diameter between groups (*p* = 0.16). From day 56 of age and until the end of the study, LP birds had a larger cloacal cleft diameter than SP birds (all *p* ≤ 0.05).

## Spatial orientation abilities

### Habituation

**Figure 4:**
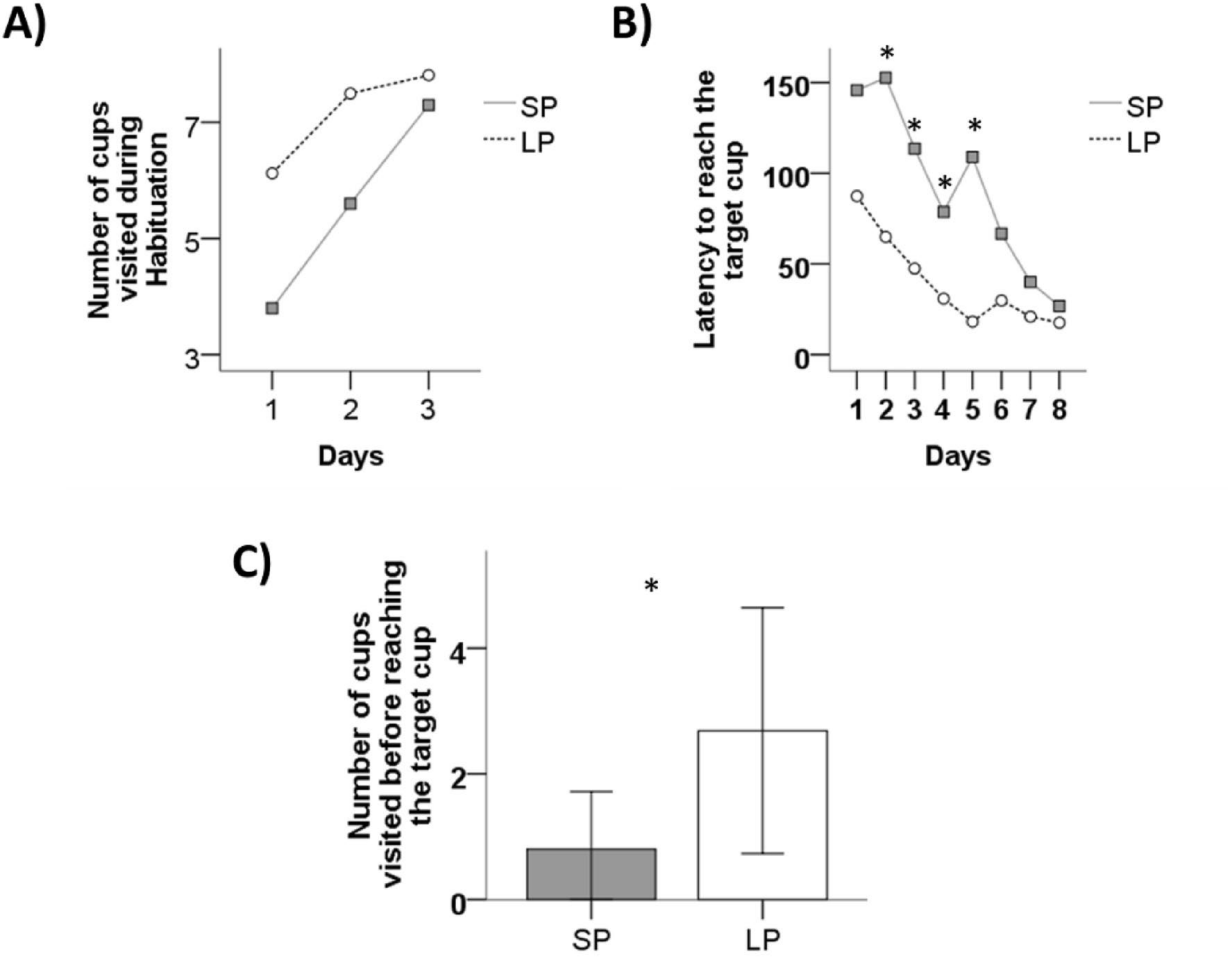
Effects of photoperiod on spatial orientation performances. (**A**) Number of visited cups over days of habituation for quail exposed to short (SP) and long (LP) photoperiod. (**B**) Latency to visit the location of the target cup (in seconds) over training days for SP and LP birds. (**C**) Number of cups visited before reaching the target cup during the probe trial for SP and LP birds. *p < 0.05. Data are presented as estimated marginal means (**A**). Data are presented as mean ± S.D. (**B**,**C**).

The number of cups visited by both SP and LP birds increased over days (effect of days: F_53.936, 3.282_ = 16.434, *p* < 0.001, ηp^2^ = 0.406; Fig. 4A), indicating that individuals from both photoperiod groups became habituated to the task. However, LP birds visited more cups overall than SP birds (effect of photoperiod group: F_1, 24_ = 7.817, *p* = 0.01, ηp^2^ = 0.246; effect of days × photoperiod group: F_7.085, 3.282_ = 2.159, *p* = 0.139, ηp^2^ = 0.083).

#### Training

During training, the latency to find the unique target cup decreased significantly across training days (effect of days: F_3.944, 94.646_ = 17.434, *p* < 0.001, ηp^2^ = 0.418). However, this decrease was significantly different between SP and LP birds (effect of photoperiod group: F_1, 24_ = 12.823, *p* = 0.02, ηp^2^ = 0.348; effect of days x photoperiod group: F_3.944, 94.646_ = 3.233, *p* = 0.016, ηp^2^ = 0.119, Fig. 4B). Post-hoc analyses revealed that while SP and LP birds did not differ on the first day of training (*p* = 0.052), SP birds were slower to reach the target cup compared to LP birds from the second to the fifth day of training (all *p* < 0.05). In contrast, these differences were no longer present in the last three days of training (all *p* > 0.05).

There were no differences between SP and LP birds for the number of cups visited before reaching the target cup over the last two days of training (effect of days: F_1, 24_ = 0.117, *p* = 0.736, ηp^2^ = 0.005; effect of photoperiod group: F_1, 24_ = 0.667, *p* = 0.422, ηp^2^ = 0.027; effect of days x photoperiod group: F_1, 24_ = 0.893, *p* = 0.354, ηp^2^ = 0.036), confirming that birds from both treatments were at the same learning level by the end of the training phase.

#### Probe trial

During the probe trial, there were no differences in the latency to reach the target cup between SP and LP quail (SP quail: 10.40 s ± 3.05 s, LP quail: 15.62 s ± 2.41 s, U = 54.54, *p* = 0.18). However, SP quail did fewer cup visits than LP quail before reaching the target cup (SP: 0.8 ± 0.29 visits, LP: 2.68 ± 0.48 visits, U = 38, *p* = 0.023, Fig. 4C).

### Behavioral reactions

**Figure 5:**
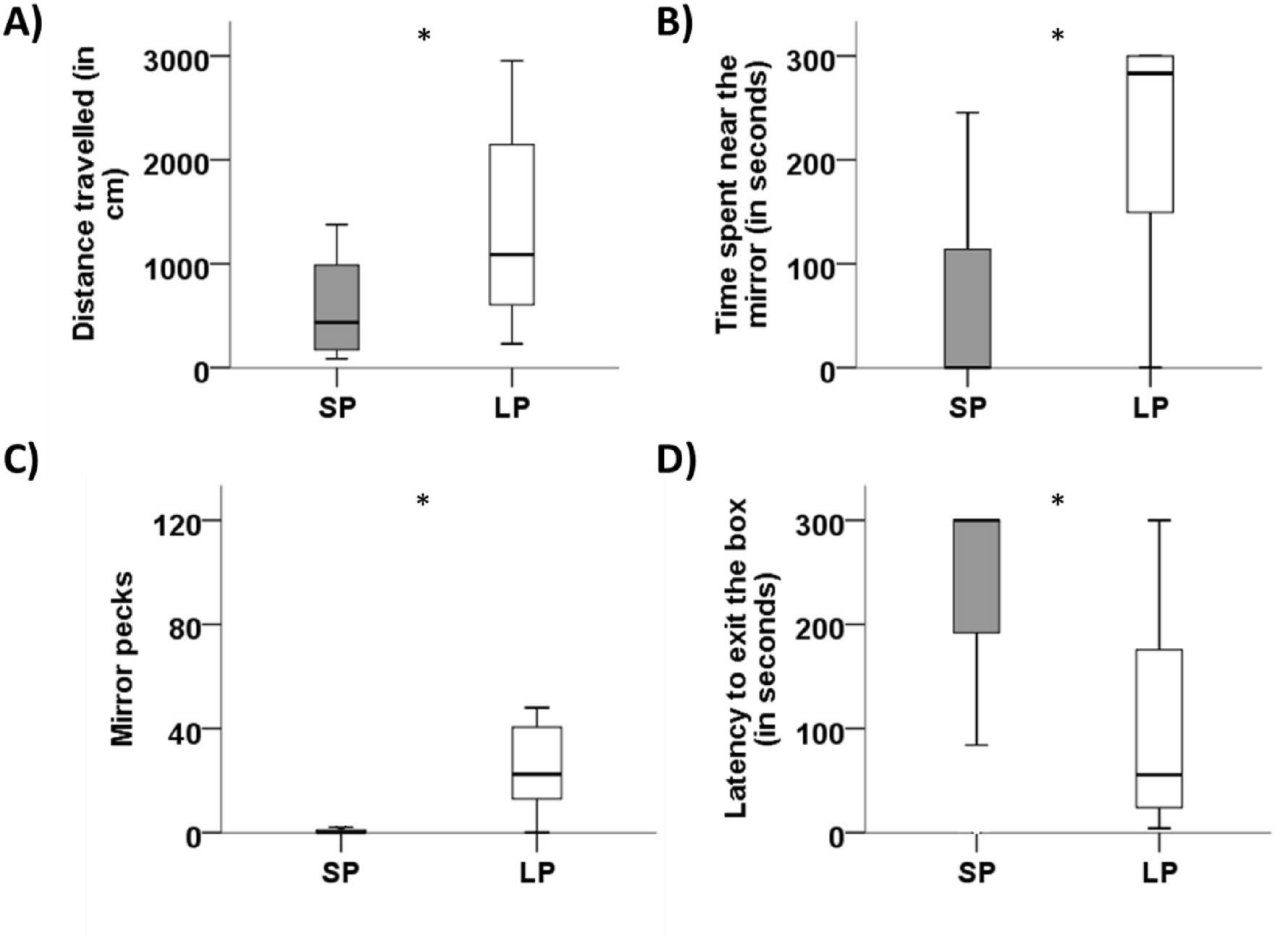
Effect of photoperiod on quail behavior. (**A**) Median distance travelled within the open field arena for quail exposed to short (SP) and long (LP) photoperiod. (**B**) Median time spent near the mirror (in seconds), during the mirror test, for SP and LP birds. (**C**) Median mirror pecks, during the mirror test, for SP and LP birds. (**D**) Latency to exit the black box compartment (in seconds), during the emergence test, for SP and LP birds. *p < 0.05. Data are presented as the 25th percentile, the median (the 50th percentile), and the 75th percentile.

In the open field test, SP quail travelled a shorter distance than LP quail (SP: 575.05 cm ± 113.51 cm, LP: 1408.74 cm ± 224.25 cm, U = 51, *p* = 0.003, Fig. 5A). SP birds also spent less time near the mirror and pecked it less than LP birds (SP quail: 56.65 s ± 21.47 s, LP quail: 232.90 s ± 23.78 s, U = 23, *p* < 0.001, Fig. 5B; SP quail: 0.93 ± 0.52, LP quail: 28.06 ± 6.83, U = 23, *p* < 0.001 Fig. 5C, respectively). Finally, SP birds took longer to emerge from a dark box into a lighted environment than LP birds (SP quail: 232.37 s ± 30.52 s, LP quail: 99.81 s ± 24.42 s, U = 55, *p* = 0.004, Fig. 5D).

### Hippocampal cell proliferation and maturation/migration of young cells

**Figure 6:**
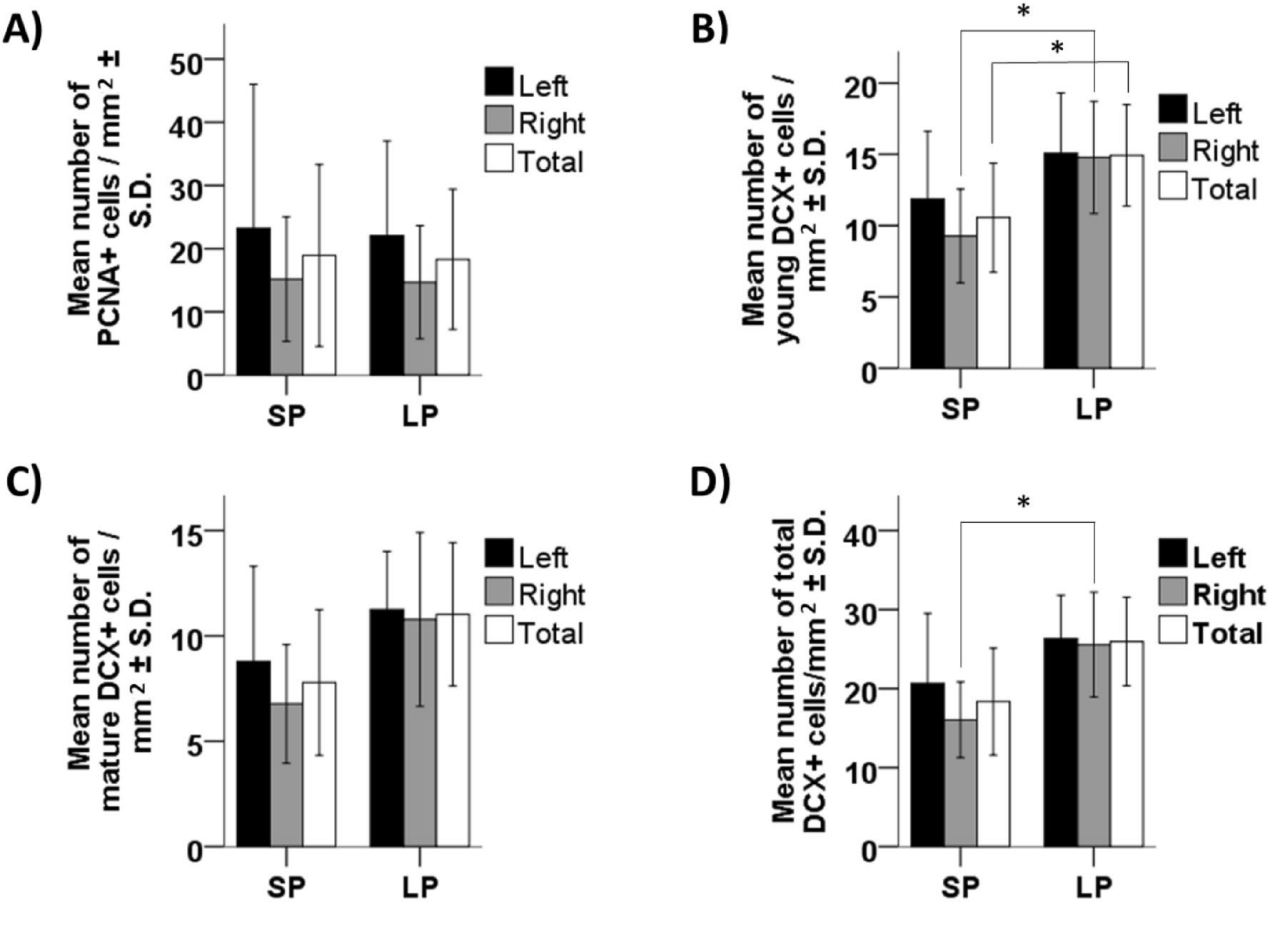
Effect of photoperiod on quail hippocampal neurogenesis. (**A**) Density of PCNA-immunoreactive cells in the hippocampus (left, right, and total) for quail exposed to short (SP) and long (LP) photoperiod. (**B**) Density of DCX young-immunoreactive cells in the hippocampus (left, right, and total), for SP and LP birds. (**C**) Density of mature-immunoreactive DCX cells in the hippocampus (left, right, and total), for SP and LP birds. (**D**) Density of young and mature-immunoreactive DCX cells in the hippocampus (left, right, and total), for SP and LP birds. *p < 0.05. Data are presented as mean ± S.D.

No significant difference was found for PCNA-immunoreactive cell density in the hippocampus between groups (Left: U = 12, *p* = 1; Right: U = 12, *p* = 1; Total: U = 10, *p* = 0.759, Fig. 6A).

The density of young-immunoreactive DCX cells in the right side and both sides combined (right + left) of the hippocampus was significantly lower in SP than in LP birds (Left: U = 12, *p* = 0.429; Right: U = 3, *p* = 0.017; Total: U = 5, *p* = 0.046, Fig. 6B). Conversely, no differences were found for the “mature-immunoreactive” DCX cells between groups (Left: U = 8, *p* = 0.146; Right: U = 6, *p* = 0.071; Total: U = 7, *p* = 0.102, Fig. 6C).

The combination of young and more mature DCX revealed a difference between groups for the right hippocampus (LP > SP), but not for the left hippocampus, nor for both sides combined (Left: U = 11, *p* = 0.354; Right: U = 3, *p* = 0.018; Total: U = 7, *p* = 0.104, Fig. 6D).

## Discussion

We investigated how photoperiod influences behavioral, cognitive, and neurobiological parameters in captive male quail. To do so, birds were subjected to two photoperiod treatments, a long photoperiod (16 h light/day) and a short photoperiod (8 h light/day), and then tested under different settings. Our findings demonstrate that photoperiod profoundly impacts how birds perceive and treat spatial information during a spatial orientation task. LP birds were more exploratory than SP birds in these tasks as they displayed more cup visits during habituation and a shorter latency to reach the target cup during intermediate training days. However, SP birds were found to be more performant, as they did fewer error visits than LP birds during the probe trial, which suggest improved spatial orientation. Differences were also found at behavioral and neurobiological levels. SP seemed to increase the emotional reactivity as quail of this group were less exploratory and less aggressive than LP birds. Finally, SP appears to reduce maturation/migration of young hippocampal cells, but not cell proliferation, as the density of DCX cells was lower in SP than in LP birds. These results clearly illustrate that photoperiod has profound effects upon various aspects of quail physiology, which go well beyond the “classical” impact upon the reproductive system, responsible for the timely control of seasonal breeding in this species. These effects also encompass modifications of behavioral traits, which taken together, likely contribute to the overall photoperiod-specific phenotype.

As expected, quail exposed to SP showed a significant regression of their reproductive system, as assessed indirectly through cloacal cleft diameter, and a decrease (or blunted increase) of their body mass. The smaller cloacal cleft diameter in SP birds compared to LP birds indicates that our animals have integrated the photoperiodic message, which is consistent with prior data in this species^21,30,43,44^. Taking into account the diurnal phenotype of quail, the simplest explanation for the body mass decrease would be that LP quail simply had more time to feed than SP quail. However, the contrasting behavioral differences over different behavioral tests between LP and SP birds suggest a more complex alternative scenario. Indeed, SP seems to enhance emotional reactivity, which might impact feeding patterns^45^. Interestingly, similar findings have also been reported in rats, which display marked nocturnal activity^46^.

The habituation and training curves during the spatial orientation task revealed that both groups were able to learn the task. However, LP quail visited more cups during habituation and were quicker to reach the target cup from the second to the fifth days of training. This difference was no longer present during the last three days of training. These results indicate that SP exposure may slow down spatial learning, either through cognitive impairment or through “non-cognitive” ways, for instance through reduction of exploration tendencies so that animals are not in contact with what is to be learned^47^. Our results are in line with current mammalian literature as adult male white-footed mice (*Peromyscus leucopus*) under SP also showed longer latencies to find the location of a submerged platform in the water maze^11^.

Probe trial performance of SP birds revealed that, although the latency to find the target cup was equivalent between groups, the number of cups visited before reaching the target cup was fewer for SP than for LP birds. Therefore, these results do not support that SP birds present some form of cognitive impairment. However, it is noteworthy that though learning is apparently delayed (during habituation and training), SP-exposed quail might orientate themselves and use spatial information in a more efficient way than LP quail.

Overall, SP increased emotional reactivity since SP birds were less exploratory, less aggressive, and less bold than LP birds. These results concur with our own data obtained using quail lines selected for > 30 years on phenotypic traits pertinent to either high or less emotionality traits. Highly emotional quail are more fearful, but also more accurate in spatial tasks^26^. Similarly, our studies on domestic free-ranging chickens suggest that individuals displaying little exploratory activity are endowed with better cognitive performance than more exploratory individuals^31,36,48^. Combining behavioral and cognitive results, some authors hypothesized that a trade-off between speed and accuracy might exist^49^. Even though animals with higher exploratory activity and increased aggressiveness may be quicker to explore their surroundings, this exploration might be rather superficial, leading to a superficial spatial map. In contrast, animals with lower exploratory activity and lowered aggressiveness would be more sensitive to environmental cues and less impulsive, taking less risks and also creating a more complex mental spatial map^49^. Our results from both cognitive and behavioral tests seem to fit this pattern and therefore support this trade-off hypothesis.

Since spatial orientation abilities depend, at least in part, upon the hippocampus, we also investigated the impact of photoperiod on neurogenesis in this brain region, through the assessment of cell proliferation and cell maturation/migration (using PCNA and DCX as markers). Photoperiod did not appear to modulate cell proliferation in the quail hippocampus. These results contrast with findings of several prior studies, which found increased cell proliferation in the hippocampus, in birds and mammals^6,14,15,50,51^. Several non-mutually exclusive explanations for these seemingly conflicting findings may be envisaged, including the role of confounding factors. Captivity-induced stress may be one of these confounding factors, as previous studies that tested birds captured in the wild failed to dissociate the impact of photoperiod from that of stress^15^. Then, the temporal sequence in which cognitive tests were performed may also be of importance as it is known that cognitive tests can influence neurological parameters, which may in turn modify the net output of subsequent tests^52,53^. During our experiment, hippocampal neurogenesis was investigated only after the cognitive tests and this may have interacted with photoperiod in ways that are beyond the scope of our investigation. Future studies should take these different confounding effects into account to better characterize the effects of photoperiod on neurobiology, with particular respect to hippocampal neurogenesis.

An impact of photoperiod on cell maturation/migration was confirmed, with a LP-increase in the number of DCX positive neurons mostly observed in the right hippocampus. This is consistent with the observation that the right hippocampus is strongly implicated in the treatment of spatial information in birds^54^. The modest photoperiod-induced alteration of neurogenesis reported here, with lowered cell maturation/migration in the right hippocampus of SP-exposed birds, might seem counterintuitive since SP birds performed better than LP birds in the spatial orientation task. However, the hippocampus is not only involved in spatial orientation/memory functions, but may also act as an emotion regulator. Indeed, a lesion or inactivation of the ventral hippocampus causes a reduction in the anxiety levels/emotional reaction of individuals^55–57^. Finally, there is no strong rationale to infer that an increase in proliferation necessarily translates into an improved function of the brain region considered.

It is important to bear in mind that male quail used in our experiments were intact, i.e., not castrated. This makes it impossible to dissociate the impact of photoperiod per se, if any, from that mediated by sex steroid hormones through the photoperiodic control of the hypothalamo-pituitary axis^21,58,59^. Studies in white-footed mice show that SP-exposed castrated animals supplemented with testosterone perform better in learning and spatial orientation tests than animals placed on long photoperiod, while injecting testosterone into LP-exposed castrated animals does not affect performance^60^. These findings underscore that part of the photoperiodic response in mice is mediated through sex steroid but do not exclude participation of sex steroid-independent mechanisms/circuits. It would be valuable to perform similar experiments in quail to disentangle the complex interaction between photoperiod and sex steroid status.

Another important limitation of our experiments is the fact that we used only males, instead of investigating the effect of photoperiod on both sexes. As results may vary between males and females, future studies should privilege the investigation of both sexes to better understand the interaction between photoperiod and sex, but also how different sex hormones interact with photoperiod.

In conclusion, our study reveals that photoperiod not only regulates breeding, but also acts as a potent modulator of multiple complex cognitive functions in quail. Exposure to SP modulates spatial orientation abilities and intensifies emotional responses. This correlates with modest changes in hippocampal neurogenesis, which suggests that plasticity mechanisms of the brain may be involved in the observed changes. To establish the role of sex steroids in the contrasted phenotypes of LP and SP quail will require further experiments. We surmise that coordinated changes of multiple behavioral, cognitive and physiological functions are all parts of an integrated seasonal program, which represents a key adaptative strategy of animals to their environment.

## Supplementary Information

Supplementary Information.

## Data Availability

All data generated or analyzed during this study are included in this published article and its supplementary information files.
